# Systematic literature review of use of blood glucose monitoring in phase III clinical studies of insulin analogs

**DOI:** 10.1186/s12902-016-0102-1

**Published:** 2016-05-04

**Authors:** Kaisa Miikkulainen, Antonio Caruso, Oliver Mast, Rongrong Zhang, Oleg Borisenko

**Affiliations:** Synergus AB, Djursholmsvägen 20C, Danderyd, 18233 Sweden; Roche Diabetes Care GmbH, Sandhofer Strasse 116, Mannheim, 68305 Germany; Roche Diagnostics GmbH, Sandhofer Strasse 116, Mannheim, 68305 Germany

**Keywords:** Blood glucose self-monitoring, Diabetes mellitus, Insulin analogues, Phase III trials, Safety, SMBG

## Abstract

**Background:**

Safe and effective insulin therapy for diabetes mellitus requires initial dose titration and regular adjustments based on blood glucose (BG) monitoring. Our objective was to explore the use of BG measurement in phase-III clinical studies of insulin analogs. These studies provide safety and efficacy information for regulatory authorities and are the basis for insulin analog regulatory approval.

**Methods:**

A systematic review of phase-III studies of rapid-acting insulin analogs (insulin lispro, insulin aspart and insulin glulisine) and pre-mixed insulin analogs (biphasic insulin aspart and insulin lispro mix) was conducted. Studies were identified using manufacturers’ databases. Search for reports was performed in Medline and registry of clinical trials (clinicaltrials.gov). The European Medicines Agency was contacted to provide Clinical Study Reports.

**Results:**

Forty-five studies were included. Regular BG measurements were reported in 100 % of the studies and were performed by either self-monitoring of blood glucose (SMBG) alone in 84 %, laboratory alone in 7 %, and both SMBG and laboratory in 9 % of studies. In total, 93 % of the studies reported SMBG. Most studies (91 %) reported insulin therapy adjustments based on BG measurements.

**Conclusions:**

The findings suggest that BG monitoring and specifically SMBG are co-dependent technologies with insulin analogs. BG measurement is used in most phase-III registration studies for establishing safe and efficacious insulin administration and is recommended in the insulin labels. The indispensable role of SMBG in treatment of insulin-dependent patients should receive attention from health care payers to assess and reimburse SMBG along with insulin to avoid adverse events from inappropriate insulin administration and associated costs.

## Background

Multiple stakeholders, including regulatory authorities, clinicians, payers (commissioners) and health technology assessors, are involved in market introduction and management pathways for health technologies. From the perspective of these stakeholders, monitoring of blood glucose (BG) in insulin-dependent diabetes has strong global support from clinical societies [1–6]. The American Diabetes Association guidelines specify that “major clinical trials of insulin-treated patients that demonstrated the benefits of intensive glycemic control on diabetes complications have included self-monitoring of blood glucose (SMBG) as part of multifactorial interventions, suggesting that SMBG is a component of effective therapy” [1]. Regulatory authorities have approved the use of blood glucose meters for use with insulin products and have outlined instructions for the measurement of BG [7]. Health technology assessments, although rarely focused on SMBG in insulin-dependent diabetes, have also been in general supportive of BG monitoring [8,9]. Lack of understanding of the role of BG monitoring in the safe and effective use of insulin leads to restrictions or no coverage of SMBG in some developing countries, while reimbursement for insulin treatment is provided [10]. According to Czupryniak et al. [11], access to SMBG is limited especially in Central and Eastern European countries. In a survey conducted in 47 countries by the International Diabetes Federation, existing limitations for access to SMBG were also confirmed [12]. Limitations usually do not have rational ground and can mainly be explained by the absence of a consistent reimbursement system. Another potential reason for restrictions of SMBG is the lack of randomized controlled trials (RCTs) for comparing the management of insulin-dependent diabetes with and without SMBG. RCTs are considered as a “gold standard” for assessment of safety and efficacy of health care interventions [13], although their role in assessment of effectiveness of SMBG as stand-alone technology can be reasonably questioned. The role of SMBG in insulin-dependent diabetes has not been studied in RCTs and does not require validation in comparative studies, as it would be considered unethical to allocate patients to insulin without the ability to determine and adjust dose based on BG measurements. Another contributing factor to complexity of assessment of efficacy of SMBG is that is it a diagnostic measure, not a treatment intervention.

SMBG and the use of insulin are ultimately linked together for an effective and safe therapy. Regulatory approval of insulin has been established in phase III clinical studies [14]. Because BG is an essential component of these foundational studies of the safety and efficacy of insulin treatment, it provides additional arguments for the use of SMBG, and thus confirms the co-dependent nature of SMBG and insulin treatment. Therefore, it is necessary to consider them jointly in evidence evaluations and reimbursement decision making, and BG monitoring should be mentioned as a part of the intervention in the studies on insulin analogs.

In a preliminary review of phase III clinical studies of insulin analogs, it was found that in only 12 of the 57 study synopses analyzed, dose adjustments were made according to BG measurements [7]. In addition, full text reports were not available for review in most of the studies. Our study aimed to explore this area further, and we hypothesized that phase III registration studies of commonly used insulin analogs (biphasic insulin aspart, insulin aspart, insulin glulisine, insulin lispro and insulin lispro mix) include BG monitoring and consequent regular dose adjustment as an essential element of the research protocol. The resulting impact on the treatment outcomes is thought to be a joint effect of the co-dependent technologies.

## Methods

A systematic literature review was performed in multiple steps. First, a systematic search for phase III clinical studies was performed on the manufacturers’ websites (Novo Nordisk A/S, Eli Lilly and Company, Sanofi S.A.). The rapid-acting insulin analogs included insulin aspart (NovoRapid®), insulin glulisine (Apidra®) and insulin lispro (Humalog®). Pre-mixed insulin analogs included biphasic insulin aspart (Novo Mix®) and insulin lispro mix (Humalog Mix®). All searches were conducted in December 2013. Second, Clinical Study Reports were requested from the European Medicines Agency (EMA), according to Regulation (EC) No 1049/2001. Third, an additional search for full-text publications was performed in Medline and Medline In-Process bibliographic databases using the clinicaltrials.gov identification number (clinical trial ID) or the manufacturer’s study ID. If no full-text publication was identified in the bibliographic databases, a search was performed in the registry of clinical studies clinicaltrial.gov. Finally, a study synopsis or summary was retrieved from the manufacturers’ websites. Only prospective phase III clinical trials of insulin analogs that reported clinical outcomes were included, as insulin analogs (in comparison with human insulin) are more recently available and have followed the most recent regulatory procedures.

Data collected included country of origin, design of the study, sample size, mean age, type of diabetes, duration of diabetes, proportion of males, use of insulin before enrollment into study, target glycated hemoglobin (HbA1c) in the inclusion and exclusion criteria, mean HbA1c, use and purpose of BG measurement, method of insulin dose adjustment, structuration of SMBG, provision of education/training for SMBG, and monitoring of compliance in SMBG. The data were extracted by one reviewer and validated by a second reviewer. Any disagreements were resolved by consensus. No statistical hypothesis was tested. The main outcomes included frequency of use of BG monitoring and use of BG monitoring for insulin dose adjustment. Summary statistics were provided using SPSS version 20 (IBM Corp., Armonk, New York, USA). Study did not require ethical approval, as it did not include any patient’s data.

## Results

### Clinical studies search

**Fig. 1:**
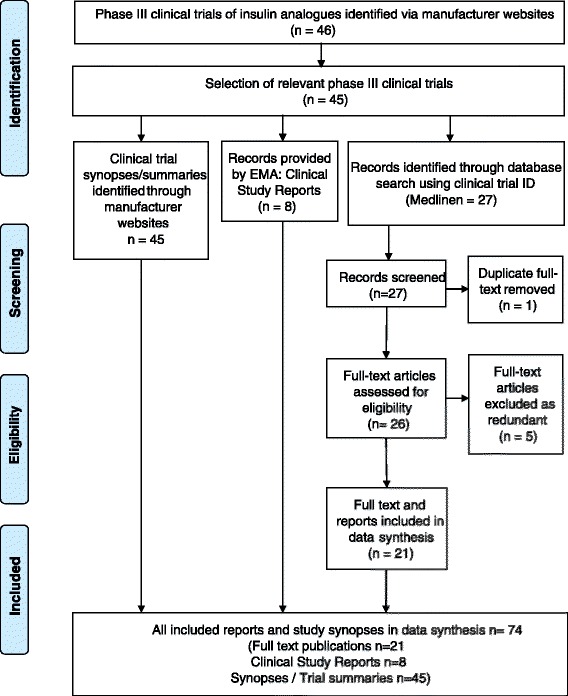
Selection of phase III trials and subsequent full-text publications (adapted from PRISMA [21])

Altogether 46 phase III clinical trials were identified in the manufacturers’ websites. One retrospective study (NCT00410033) was excluded from analysis, as it did not meet inclusion criteria. The database search in Medline provided 27 full-text articles, one of which was identified as a duplicate [15] and five as redundant publications [16–20]. All five redundant publications were considered less informative than the main published phase III clinical study in question. EMA provided eight out of the 45 requested extracts of Clinical Study Reports of the phase III insulin analog studies. The majority of the Clinical Study Reports were not held by EMA. The analog reports which were provided consisted of the following: insulin glulisine (*n* = 1), biphasic insulin aspart (*n* = 3) and insulin lispro (*n* = 4). For three studies, both the Clinical Study Reports and full-text publications were available, but for five studies, only the Clinical Study Report was available. A detailed description of the selection process as adapted from the Preferred Reporting Items for Systematic Reviews and Meta-Analyses (PRISMA) framework [21] is presented in Fig. 1.

### Description of included studies

The characteristics of the included studies are described in Table 1. Of the identified phase III studies, 62 % (*n* = 28) were rapid-acting insulin analog studies and 38 % (*n* = 17) were pre-mixed insulin analog studies. Of the rapid-acting analogs, 29 % (*n* = 8) were insulin aspart, 57 % (*n* = 16) were insulin glulisine and 14 % (*n* = 4) were insulin lispro. Of the pre-mixed insulin analog studies, 71 % (*n* = 12) were biphasic insulin aspart studies and 29 % (*n* = 5) insulin lispro mix studies.

**Table 1:** Characteristics of the included study reports

Insulin analog type	Insulin analog	Clinical trial ID	EMA report	First author of full-text article	Year	Design	Sample size	Mean age of total	Population	DM type	Mean duration of DM in years	Male (%)	Mean HbA1c (%) at baseline
Rapid-acting	Insulin aspart (Novo Rapid)	NCT01388361	N	Mathieu [29]	2014	RCT	413	61.3	A	II	11.8	64	7.3
		NCT00097071	N	Weinzimer [30]	2008	RCT	299	13.1	C	I	6.1	48	8.1
		NCT00604656	N	None	2004	RCT	241	40.2	A	I	13.6	66	7.8
		NCT00071448	N	None	2006	RCT	378	11.6	C	I	4.6	52	8.3
		NCT01486940	N	Hermansen [27]	2004	RCT	644	39.1	A	I	15.2	63	8.4
		NCT00065130	N	Pettitt [31]	2007	RCT	27	30.7	A	GDM	–	0	5.2
		NCT00832182	N	None [32]*	2002	Cohort	75	–	A	I	–	–	–
		NCT01707134	N	Home [33]	2006	RCT	753	38.8	A	I	15.2	58	–
	Insulin glulisine (Apidra)	NCT00467376	N	None	2009	RCT	484	–	A	I, II	10.3	–	8.6
		NCT00115570	N	None	2009	RCT	572	12.5	C	I	–	–	–
		NCT00290979	N	None	2006	RCT	267	–	A	I	–	–	–
		NCT00290927	N	None	2005	RCT	393	–	A	II	–	–	–
		NCT00135057	Y	Bergenstal [34]	2008	RCT	270	55.1	A	II	13.0	44	8.2
		NCT00135096	N	Ratner [35]	2011	RCT	716	53.8	A	II	14.0	44	8.4
		NCT00174668	N	None	2008	RCT	310	–	A	II	–	–	–
		NCT00546702	N	None	2005	CS	140	–	A	I	–	–	–
		NCT00545337	N	None	2007	CS	60	34.7	A	I	12.7	45	–
		NCT00174642	N	None	2009	RCT	464	58.5	A	II	–	45	9.1
		NCT00272012	N	None	2007	RCT	393	–	A	II	–	–	–
		NCT00135941	N	Testa [36]	2012	RCT	388	54.0	A	I, II	16.1	47	7.8
		NCT00135083	N	Davidson [37]	2011	RCT	343	–	A	II	–	5	–
		NCT00272064	N	Del Prato [38]	2012	RCT	241	58.3	A	II	10.9	52	8.9
		NCT00271284	N	Renard [39]	2011	RCT	135	46.8	A	I	18	61	7.1
		NCT00397553	N	None	2009	CS	104	35.0	A	I	25.3	–	8.8
	Insulin lispro (Humalog)	F3Z-MC-IOAE	Y	None	1994	RCT	98	24.4	M	I	0.2	61	–
		F3Z-MC-IOAF	Y	None	1994	RCT	377	56.1	A	II	7.8	56	–
		F3Z-MC-IOAG	Y	None	1993	RCT	1037	33.4	M	I	12.1	58	8.5
		F3Z-MC-IOAH	Y	None	1993	RCT	777	58.6	A	II	12.6	54	8.9
Pre-mixed	Biphasic insulin aspart (Novo Mix)	NCT00476437	N	None	2008	RCT	81	62.3	A	II	18.3	54	7.8
		NCT00318786	N	Kadowaki [40]	2010	RCT	289	62.5	A	II	15.7	59	8.5
		NCT00313001	N	None	2007	RCT	372	52.6	A	II	–	48	10.2
		NCT00184574	N	Cucinotta [41]	2009	RCT	603	60.5	A	II	–	44	8.9
		NCT00097877	N	Bergenstal [42]	2009	RCT	372	52.6	A	II	–	48	10.2
		NCT00184600	N	Holman [18]	2009	RCT	708	61.7	A	II	9	64	8.5
		NCT00097279	Y	Raskin [43]	2007	RCT	230	53.8	A	II	8.8	42	8.1
		NCT00564668	N	None	2005	RCT	126	62.2	A	II	16.4	62	7.2
		NCT00612599	N	Parkner [44]	2010	RCT	75	60.4	A	II	11.7	70	7.5
		NCT00617565	Y	None	2004	RCT	219	55.8	A	I, II	11.1	56	8.8
		NCT01467375	N	None [45]*	2005	CS	89	65.1	A	II	15.2	65	8.1
		NCT01467323	Y	Boehm [46]	2002	RCT	294	54.0	A	I, II	15.3	58	8.3
	Insulin lispro mix (Humalog Mix)	NCT00191581	N	Gao [47]	2008	RCT	120	55.7	A	I, II	11.4	40	8.1
		NCT00551356	N	None	2003	RCT	53	50.4	A	II	6.5	57	–
		F3Z-JE-IOMO	N	None	2003	RCT	215	55.9	A	I, II	15.0	58	–
		F3Z-MC-IONA	N	None	2006	RCT	106	52.4	A	I, II	10.4	52	–
		NCT00036504	N	None	2007	RCT	105	54.9	A	II	8.9	62	8.6

*Abbreviations*: *EMA* European Medicines Agency; design: *RCT* randomized control trial, *CS* case series study; population: *A* adult, *C* child and/or adolescent, *M* mixed

*DM* diabetes mellitus; type: I diabetes mellitus type I, II–diabetes mellitus type II, GDM-gestational diabetes; HbA1c-glycated hemoglobin

*Unpublished study. Reference provides additional information

The majority (*n* = 40, 89 %) of the included studies were RCTs. In total 60 % (*n* = 27) were single-country studies conducted in USA (*n* = 9, 20 %), Japan (*n* = 5, 11 %), China (*n* = 3, 7 %), Russia (*n* = 2, 5 %), and 1 (*n* = 1, 2 %) each in Germany, Denmark, Italy, France, Mexico, the Netherlands and Ukraine. There were 29 % (*n* = 13) of the studies conducted in multiple countries; the location was not specified in 14 % (*n* = 6) of the studies. The median number of patients in the studies was 280 (interquartile range 113–403).

Type 1 diabetes mellitus (DM) patients were included in 31 % (*n* = 14) and type 2 DM patients in 51 % (*n* = 23) of the studies. A mixed population consisting of patients with both type 1 and 2 DM was identified in 16 % (*n* = 7) of the studies. One study included patients with gestational DM only.

In 89 % (*n* = 40) of the studies, only adults were included, and in 7 % (*n* = 3) only children and/or adolescents were included. In two (4 %) studies, the patient population was mixed where both adults and children were included. Mean age of patients in all studies was 48.3 (standard deviation [SD] 14.6) years. Males constituted on average 53 % of all study participants. The mean duration of diabetes prior to enrolment was 12.2 (SD 4.6) years and insulin therapy had been initiated prior to enrolment in 78 % of the studies. Patients had not received insulin therapy prior to enrolment in 13 % of the studies. The mean level of HbA1c at baseline was 8.3 % (SD 0.9 %).

### BG monitoring in the included studies

#### BG monitoring

**Fig. 2:**
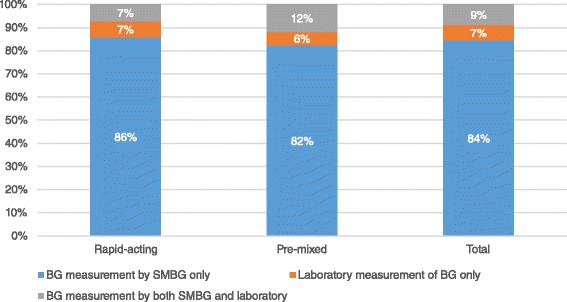
Proportion of phase III clinical trials that reported regular BG monitoring per insulin analog type

A summary of the results is presented in Table 2. Regular monitoring of BG was reported in 100 % (*n* = 45) of the studies as shown in Fig. 2.

**Table 2:** Review of phase III insulin analog clinical trials

	Rapid-acting insulin analogs			Pre-mixed insulin analogs		Total		
	Insulin aspart (Novo Rapid)	Insulin glulisine (Apidra)	Insulin lispro (Humalog)	Biphasic insulin aspart (Novo Mix)	Insulin lispro mix (Humalog Mix)	Rapid-acting	Pre-mix	All insulin analog studies
Total number of trials (n)	8	16	4	12	5	28	17	45
*All insulin analog studies (n = 45)*								
Proportion of trials reporting BG measurement (%)	100 %	100 %	100 %	100 %	100 %	100 %	100 %	100 %
*Reported BG by SMBG, laboratory or both (n = 45)*								
Reported BG measurement by SMBG only (%)	100 %	82 %	75 %	83 %	80 %	86 %	82 %	84 %
Reported BG measurement by laboratory measurement only (%)	0 %	12 %	0 %	0 %	20 %	7 %	6 %	7 %
Reported BG by both SMBG and laboratory measurements (%)	0 %	6 %	25 %	17 %	0 %	7 %	12 %	9 %
*Reported regular insulin dose adjustments (using BG measurements, n = 45)*								
Reported dose adjustments according to BG measurement (%)	87.5 %	100 %	100 %	91.6 %	60.0 %	96.4 %	82.4 %	91.1 %
*Reported regular dose adjustments by patient or physician (using BG measurements, n = 45)*								
Reported dose adjustments by patient (%)	25.0 %	43.7%	0 %	41.7 %	0 %	32.1 %	29.4 %	31.1 %
Reported dose adjustments by physician (%)	37.5 %	25.0 %	100 %	33.3 %	40.0 %	39.3 %	35.3 %	37.8 %
Reported dose adjustments by patient and physician (%)	0 %	6.3 %	0 %	8.3 %	0 %	3.6 %	5.9 %	4.4 %
Reported dose adjustments, not specified by patient or physician (%)	25.0 %	25.0 %	0 %	8.3 %	20.0 %	21.4 %	11.8 %	17.8 %
*Reported regular dose adjustments using SMBG measurements (n = 42)*								
Patient self-adjustments using SMBG values (%)	25.0 %	50 %	0 %	41.7 %	0 %	34.6 %	31.3 %	33.3 %
Physician adjustments using SMBG values (%)	37.5 %	28.6 %	75 %	33.3 %	50 %	38.5 %	37.5 %	38.1 %
Patient and physician adjustments using SMBG values (%)	0 %	7.1 %	0 %	8.3 %	0 %	3.8 %	6.3 %	4.8 %
Dose adjustments using SMBG mentioned but not specified whether by patient or physician (%)	25.0 %	7.1 %	0 %	0 %	25 %	11.5 %	6.3 %	9.5 %

**Fig. 3:**
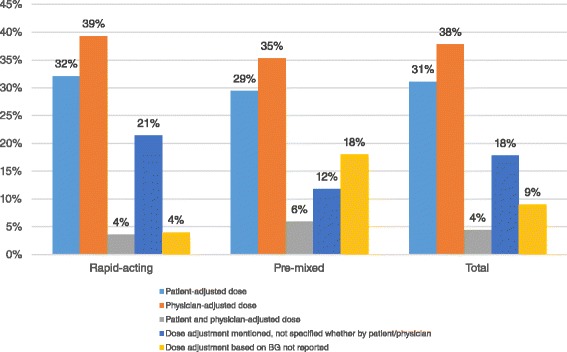
Proportion of phase III trials reporting insulin dose adjustments

Insulin doses were reportedly adjusted based on regular BG measurements in 91 % of phase III studies (Fig. 3). In total 31.1 % the studies were with patients making insulin dose adjustments using BG values. Physician-adjusted insulin dosing using BG values was reported in 37.8 % of the clinical studies that reported BG monitoring. In 4.4 % of the studies dose adjustment was reported by both patients and physicians. In 18 % of the studies, dose adjustment based on BG values was performed, but it was not specified by whom.

#### SMBG monitoring

SMBG was reported in 93 % (*n* = 42) of the phase III clinical studies. The proportions of studies using either rapid-acting or pre-mixed insulin analogs were similar in terms of those reporting SMBG. SMBG was used as the only means of BG monitoring in 84 % (*n* = 38) of the studies. Monitoring of BG using only laboratory means was reported in 7 % (*n* = 3) of the studies. Both laboratory and SMBG measurements were reported in 9 % (*n* = 4) of the studies.

With regard to insulin dose adjustments based on SMBG, 33.3 % (*n* = 14) of the phase III clinical studies reported that patients self-adjusted insulin doses based on SMBG results. The proportion of these studies using either rapid-acting (*n* = 9, 34.6 %) or pre-mixed insulin (*n* = 5, 31.3 %) was the same. Adjustment of insulin doses by physicians based on SMBG values were reported in 38.1 % (*n* = 16) of the studies. Dose adjustment by physicians based on SMBG was similar in the pre-mixed (*n* = 6, 37.5 %) and the rapid-acting insulin analog (*n* = 10, 38.5 %) studies. In two studies (4.8 %) SMBG-based dose adjustments were reported both by patients and physicians. In four (9.5 %) studies that reported dose adjustments based on SMBG, it could not be determined whether the dose adjustments were carried out by physician or patient.

Daily frequencies of SMBG measurement were reported in 83 % (*n* = 35) of the studies that reported the use of SMBG. In general, the daily frequencies varied between one to nine tests.

Training, education and provision of instructions for the proper use of SMBG were reported in 55 % (*n* = 23) of the phase III studies where SMBG was reported. Monitoring the proper use of SMBG was reported in 64 % (*n* = 27) of the studies that reported SMBG measurements. The monitoring was frequently done by regular checks via telephone and by inspecting patient diaries.

## Discussion

To our knowledge, the present study is the first to systematically assess the role of SMBG in phase III clinical studies. It confirms the role of BG measurement as an essential component of safe and effective insulin therapy. We found that in 100 % of phase-III clinical studies of analog insulins, BG monitoring was regularly used. SMBG was the most prevalent monitoring option and was reported in 93 % of the studies.

We also found that in the majority of the studies insulin therapy was regularly adjusted based on the BG measurements. Therefore, the safety and efficacy of insulin use requires guidance for patients in the use of BG measurements and the corresponding dose adjustments. Of the studies reporting BG use, 31 % included insulin dose self-adjustment by patients, 38 % with dose adjustment by physicians, 4 % with dose adjustment by both patients and physicians and finally 18 % in which dose adjustment was performed, but it was not reported by whom. For the other studies that did not explicitly mention insulin dose adjustment based on BG measurement, we cannot rule out that dose adjustment did not occur. However, all these studies were unpublished and no Clinical Study Reports were available. If the analysis is limited only to the studies for which either full text publication or the Clinical Study Report is available, then insulin dose adjustment based on BG measurements would be in 100 % of the studies, both for rapid-acting and pre-mixed insulins.

Our analysis also showed that only 55 % of the examined studies report that training, education or provision of instructions about the use of SMBG took place. We believe that this relatively low value reflects incomplete reporting in the studies, rather than a lack of provision of training. Studies have demonstrated that structured testing can enhance the value of SMBG [22–24]. Structured testing means measuring BG at specific times or in relation to activities in order to best evaluate the effect of therapy, diet and physical activity on the BG level [25]. In a review of the European Public Assessment Reports for insulin analogs, it was revealed that unspecific and unclear recommendations for BG monitoring is occasionally provided [7]. To ensure safe and effective use of insulin, manufacturers should be encouraged to provide clearer recommendations for patients and medical professionals.

This study contributes to the understanding of the essential role of BG monitoring in insulin-dependent diabetes. Insulin is safe and effective if used only in combination with regular BG monitoring. This is true for both rapid-acting and pre-mixed insulins, the latter typically used in conventional insulin therapy. The value of BG monitoring is for providing essential information for making therapeutic choices, either by the patient or the physician. And SMBG is only helpful, if it results in therapeutic consequences. To achieve that SMBG shall be placed in the center of the disease management for diabetic patients, including appropriate training of structured testing, as mentioned above. The use of both SMBG and insulin is funded in the majority of developed countries by payers. In some developing countries, reimbursement is not available for BG meters and strips, while coverage for insulin treatment is provided. The lack of affordability or reimbursement limitations of SMBG may impair adherence to the recommended BG test frequencies and result in not achieving the required level of efficacy and safety of insulin, which could ultimately lead to adverse outcomes for the patients and an increase in cost to the health care system due to ineffective management of diabetes.

Data on the impact of non-provision of SMBG on insulin-dependent patients are limited, although some emerging evidence shows a potential serious risk of a reduction of insulin effectiveness. In China, where SMBG is not reimbursed, a study in 2011 with 10,418 patients with diabetes revealed that a high proportion of insulin-dependent patients do not have a meter at home to perform SMBG (51.6 % of patients on intensive insulin therapy, 46.6 % on conventional insulin therapy and 39.9 % on basal-supported oral therapy) [26]. In all three of these clinical groups, the average level of HbA1c was higher in patients who did not have access to a SMBG meter (e.g., HbA1c 8.1 % with SMBG vs HbA1c 9.3 % without SMBG among patients on intensive insulin therapy). In another study from India (*n* = 2250) using a similar study methodology as in the study in China of accessibility to SMBG, it was found that 41.5 % of patients on intensive insulin therapy, 54.3 % on conventional insulin therapy and 44.6 % on basal-supported oral therapy did not have a meter at home to perform SMBG [27]. The level of glycemic control was lower in patients who did not perform SMBG. In a study performed in Italy, it was demonstrated that patients using SMBG had a reduced risk of diabetes-related hospitalizations and consequently a lower overall total annual cost per patient [28]. Total annual cost was €3060 in the group of no SMBG users vs. €2738 in the group of patients using SMBG, which was mainly explained by statistically significant reduction of diabetes-related hospitalizations.

Non-coverage of SMBG in some developing countries is a good example of ‘silo budgeting’ to overcome, when different payers are responsible for provision of drugs and diagnostics/medical aids. Being disconnected, the decision-making and financial flows for drugs and medical aids may lead to a break in provision of strongly co-dependent technologies, such as with insulin and SMBG. Many countries have taken action to overcome this hurdle, which requires a holistic view of the outcomes and costs in health care from the perspective of the entire society and not just from the perspective of a single payer.

Our study has some limitations. First, our search for full text articles was limited to one bibliographic database and was performed using a clinical trial identifier, which may have led to the loss of some relevant publications. Nevertheless, we were able to answer the primary question about the use of BG monitoring from the studies examined using a combination of sources, including full text articles, study synopses and EMA reports. The non-inclusion of existing full text reports may have potentially led to underreporting of dose adjustment, based on SMBG, and to the provision for training to patients on the use of SMBG. Second, our study scope was only on registered phase III studies, which was used as a foundation for insulin analog safety and efficacy claims and consequent regulatory approval, and did not include other pre- and post-marketing studies.

## Conclusions

To conclude, the findings from this review endorse the crucial role of BG measurement in general and of SMBG specifically as a co-dependent intervention component of insulin treatment. The majority of phase III registration studies reported on the use of BG measurements for establishing safe and efficacious administration of insulin. The indispensable role of SMBG in treatment for insulin-dependent patients should receive the attention of health care payers in order to consistently assess and reimburse SMBG along with insulins to ensure the safety and effectiveness of insulin treatment.

## Ethics approval and consent to participate

Not applicable.

## Consent for publication

Not applicable.

## Availability of data and materials

All information, required to interpret results of the study is provided in the Tables 1 and 2 of the article.

## Abbreviations

BG: blood glucose
DM: diabetes mellitus
EMA: European Medicines Agency
HbA1c: glycated hemoglobin
RCTs: randomized controlled trials
SD: standard deviation
SMBG: self-monitoring of blood glucose

## References

[1] American Diabetes A. American (2014). Standards of medical care in diabetes—2014. Diabetes Care.

[2] Scottish Intercollegiate Guidelines Network (SIGN) (2010). Management of diabetes. A national clinical guideline.

[3] Working Group of the Clinical Practice Guideline on Diabetes Mellitus Type 1 (2012). Clinical practice guideline for diabetes mellitus type 1.

[4] Medical Services Commission (2010). Diabetes care.

[5] Rewers M, Pihoker C, Donaghue K, Hanas R, Swift P, Klingensmith GJ (2009). Assessment and monitoring of glycemic control in children and adolescents with diabetes. Pediatr Diabetes.

[6] Medical Services Commission. Diabetes care. Victoria (BC): British Columbia Medical Services Commission; 2010. p.17. https://www.nice.org.uk/guidance/cg15. Accessed 18 Dec 2013.

[7] Caruso A, Hilsdorf HL, Mast O (2013). Self-monitoring of blood glucose with insulin analogues: nice to have or need to have? analysis of phase III registration trials and european public assessment reports of insulin analogues. Value Health.

[8] Full reference: Canadian Optimal Medication Prescribing and Utilization Service.Optimal therapy recommendations for the prescribing and use of blood glucose test strips. Ottawa (ON): Canadian Agency for Drugs and Technologies in Health; 2009. https://www.cadth.ca/media/pdf/compus_BGTS_OT_Rec_e.pdf. Accessed 18 Dec 2013.

[9] Washington State Health Care Authority. Glucose Monitoring: Selfmonitoring in individuals with insulin dependent diabetes, 18 years of age or under. Olympia (WA): Health Technology Assessment Program; 2011. http://www.hca.wa.gov/hta/documents/glucose_monitoring_final.pdf. Accessed 18 Dec 2013.

[10] The SMBG International Working Group (2008). Self-monitoring of blood glucose in type 2 diabetes: an inter-country comparison. Diabetes res clin pract.

[11] Czupryniak L, Barkai L, Bolgarska S, Bronisz A, Broz J, Cypryk K (2014). Self-monitoring of blood glucose in diabetes: from evidence to clinical reality in central and eastern europe—recommendations from the international central-eastern european expert group. Diabetes Technol Ther.

[12] International Diabetes Federation Europe. Access to quality medicines and medical devices for diabetes care in Europe. 2013. http://www.idf.org/sites/default/files/FULL-STUDY.pdf. Accessed 18 December 2013.

[13] Higgins JPT, Green S (editors). Cochrane Handbook for Systematic Reviews of InterventionsVersion 5.1.0 [updated March 2011]. The Cochrane Collaboration, 2011. http://handbook.cochrane.org. Accessed 18 Dec 2013.

[14] European Medicines Agency (EMA). ICH Topic E 8 General Considerations for Clinical Trials. Note for guidance on general considerations for clinical trials (CPMP/ICH/291/95). 1998. http://www.ema.europa.eu/docs/en_GB/document_library/Scientific_guideline/2009/09/WC500002877.pdf. Accessed 18 Dec 2013.

[15] Weinzimer SA, Ternand C, Campbell H, Chang C-T, Becker DJ, Laffel LMB (2007). A randomized trial comparing continuous subcutaneous insulin infusion of insulin aspart versus insulin lispro in children and adolescents with type 1 diabetes. Diabetes Care.

[16] Garber AJ, Ligthelm R, Christiansen JS, Liebl A (2007). Premixed insulin treatment for type 2 diabetes: analogue or human?. Diabetes Obes Metab.

[17] Jenkins N, Hallowell N, Farmer AJ, Holman RR, Lawton J (2011). Participants’ experiences of intensifying insulin therapy during the treating to target in type 2 diabetes (4-T) trial: qualitative interview study. Diabet med : j Br Diabet Assoc.

[18] Holman RR, Farmer AJ, Davies MJ, Levy JC, Darbyshire JL, Keenan JF (2009). Three-year efficacy of complex insulin regimens in type 2 diabetes. N Engl J Med.

[19] Lindholm A, Jensen LB, Home PD, Raskin P, Boehm BO, Rastam J (2002). Immune responses to insulin aspart and biphasic insulin aspart in people with type 1 and type 2 diabetes. Diabetes Care.

[20] Pettitt DJ, Ospina P, Kolaczynski JW, Jovanovic L (2003). Comparison of an insulin analog, insulin aspart, and regular human insulin with no insulin in gestational diabetes mellitus. Diabetes Care.

[21] Moher D, Liberati A, Tetzlaff J, Altman D. The PRISMA Group (2009). Preferred Reporting Items for Systematic Reviews and MetaAnalyses: The PRISMA Statement. PLoS Med. 2009;6(7):e1000097. doi: 10.1371/journal.pmed.1000097. Epub 2009 Jul 21. 10.1371/journal.pmed.1000097 PMC2707599 19621072

[22] Polonsky WH, Fisher L, Schikman CH, Hinnen DA, Parkin CG, Jelsovsky Z (2011). A structured self-monitoring of blood glucose approach in type 2 diabetes encourages more frequent, intensive, and effective physician interventions: results from the STeP study. Diabetes Technol Ther.

[23] Polonsky WH, Fisher L, Schikman CH, Hinnen DA, Parkin CG, Jelsovsky Z (2011). Structured self-monitoring of blood glucose significantly reduces A1C levels in poorly controlled, noninsulin-treated type 2 diabetes: results from the structured testing program study. Diabetes Care.

[24] Kato N, Cui J, Kato M (2013). Structured self-monitoring of blood glucose reduces glycated hemoglobin in insulin-treated diabetes. J Diabetes Invest.

[25] Ceriello A, Barkai L, Christiansen JS, Czupryniak L, Gomis R, Harno K (2012). Diabetes as a case study of chronic disease management with a personalized approach: the role of a structured feedback loop. Diabetes Res Clin Pract.

[26] Mast O, Tan A, Zweyer A, Perridy D (2012). Usage of self-monitoring of blood glucose (SMBG) by diabetes therapy type in larger cities in china. Value Health.

[27] Hermansen K, Fontaine P, Kukolja KK, Peterkova V, Leth G, Gall MA (2004). Insulin analogues (insulin detemir and insulin aspart) versus traditional human insulins (NPH insulin and regular human insulin) in basal-bolus therapy for patients with type 1 diabetes. Diabetologia.

[28] Degli Esposti L, Saragoni S, Blini V, Buda S (2014). Effect of self-monitoring of blood glucose on glycemic control, clinical outcomes, and health care costs in diabetic patients using insulin: a retrospective analysis. Value Health.

[29] Mathieu C, Rodbard HW, Cariou B, Handelsman Y, Philis-Tsimikas A, Ocampo Francisco AM (2014). A comparison of adding liraglutide versus a single daily dose of insulin aspart to insulin degludec in subjects with type 2 diabetes (BEGIN: VICTOZA ADD-ON). Diabetes Obes Metab.

[30] Weinzimer SA, Ternand C, Howard C, Chang C-T, Becker DJ, Laffel LMB (2008). A randomized trial comparing continuous subcutaneous insulin infusion of insulin aspart versus insulin lispro in children and adolescents with type 1 diabetes. Diabetes Care.

[31] Pettitt DJ, Ospina P, Howard C, Zisser H, Jovanovic L (2007). Efficacy, safety and lack of immunogenicity of insulin aspart compared with regular human insulin for women with gestational diabetes mellitus. Diabet med : j Br Diabet Assoc.

[32] Tamas G, Marre M, Astorga R, Dedov I, Jacobsen J, Lindholm A (2001). Glycaemic control in type 1 diabetic patients using optimised insulin aspart or human insulin in a randomised multinational study. Diabetes Res Clin Pract.

[33] Home PD, Hallgren P, Usadel KH, Sane T, Faber J, Grill V (2006). Pre-meal insulin aspart compared with pre-meal soluble human insulin in type 1 diabetes. Diabetes Res Clin Pract.

[34] Bergenstal RM, Johnson M, Powers MA, Wynne A, Vlajnic A, Hollander P (2008). Adjust to target in type 2 diabetes: comparison of a simple algorithm with carbohydrate counting for adjustment of mealtime insulin glulisine. Diabetes Care.

[35] Ratner R, Wynne A, Nakhle S, Brusco O, Vlajnic A, Rendell M (2011). Influence of preprandial vs. Postprandial insulin glulisine on weight and glycaemic control in patients initiating basal-bolus regimen for type 2 diabetes: a multicenter, randomized, parallel, open-label study (NCT00135096). Diabetes Obes Metab.

[36] Testa MA, Gill J, Su M, Turner RR, Blonde L, Simonson DC (2012). Comparative effectiveness of basal-bolus versus premix analog insulin on glycemic variability and patient-centered outcomes during insulin intensification in type 1 and type 2 diabetes: a randomized, controlled, crossover trial. J Clin Endocrinol Metab.

[37] Davidson MB, Raskin P, Tanenberg RJ, Vlajnic A, Hollander P (2011). A stepwise approach to insulin therapy in patients with type 2 diabetes mellitus and basal insulin treatment failure. Endocr pract : off j Am Coll Endocrinol Am Assoc of Clin Endocrinologists.

[38] Del Prato S, Nicolucci A, Lovagnini-Scher AC, Turco S, Leotta S, Vespasiani G (2012). Telecare provides comparable efficacy to conventional self-monitored blood glucose in patients with type 2 diabetes titrating one injection of insulin glulisine-the ELEONOR study. Diabetes Technol Ther.

[39] Renard E, Dubois-Laforgue D, Guerci B (2011). Non-inferiority of insulin glargine versus insulin detemir on blood glucose variability in type 1 diabetes patients: a multicenter, randomized, crossover study. Diabetes Technol Ther.

[40] Kadowaki T, Nishida T, Kaku K (2010). 28-week, randomized, multicenter, open-label, parallel-group phase III trial to investigate the efficacy and safety of biphasic insulin aspart 70 thrice-daily injections vs twice-daily injections of biphasic insulin aspart 30 in patients with type 2 diabetes. J diabetes invest.

[41] Cucinotta D, Smirnova O, Christiansen JS, Kanc K, le Devehat C, Wojciechowska M (2009). Three different premixed combinations of biphasic insulin aspart—comparison of the efficacy and safety in a randomized controlled clinical trial in subjects with type 2 diabetes. Diabetes Obes Metab.

[42] Bergenstal R, Lewin A, Bailey T, Chang D, Gylvin T, Roberts V (2009). Efficacy and safety of biphasic insulin aspart 70/30 versus exenatide in subjects with type 2 diabetes failing to achieve glycemic control with metformin and a sulfonylurea. Curr Med Res Opin.

[43] Raskin P, Matfin G, Schwartz SL, Chaykin L, Chu PL, Braceras R (2009). Addition of biphasic insulin aspart 30 to optimized metformin and pioglitazone treatment of type 2 diabetes mellitus: the ACTION study (achieving control through insulin plus oral ageNts). Diabetes Obes Metab.

[44] Parkner T, Dyrskog SE, Laursen T, Chen JW, Mouritzen U, Brondsted L (2010). Obesity does not influence the unique pharmacological properties of different biphasic insulin aspart preparations in patients with type 2 diabetes. Diabetes Obes Metab.

[45] Davidson JA, Liebl A, Christiansen JS, Fulcher G, Ligthelm RJ, Brown P (2009). Risk for nocturnal hypoglycemia with biphasic insulin aspart 30 compared with biphasic human insulin 30 in adults with type 2 diabetes mellitus: a meta-analysis. Clin Ther.

[46] Boehm BO, Home PD, Behrend C, Kamp NM, Lindholm A (2002). Premixed insulin aspart 30 vs. Premixed human insulin 30/70 twice daily: a randomized trial in type 1 and type 2 diabetic patients. Diabet Med.

[47] Gao Y, Li G, Li Y, Guo X, Yuan G, Gong Q (2008). Postprandial blood glucose response to a standard test meal in insulin-requiring patients with diabetes treated with insulin lispro mix 50 or human insulin mix 50. Int J Clin Pract.

